# Extracellular vesicles derived from *Talaromyces marneffei* contain immunogenic compounds and modulate THP-1 macrophage responses

**DOI:** 10.3389/fimmu.2023.1192326

**Published:** 2023-06-29

**Authors:** Kritsada Pruksaphon, Artid Amsri, Patcharin Thammasit, Joshua D. Nosanchuk, Sirida Youngchim

**Affiliations:** ^1^ Department of Microbiology, Faculty of Medicine, Chiang Mai University, Chiang Mai, Thailand; ^2^ Department of Medicine (Division of Infectious Diseases) and Department of Microbiology and Immunology, Albert Einstein College of Medicine, New York, NY, United States

**Keywords:** *Talaromyces marneffei*, extracellular vesicles (EVs), THP-1 macrophages, inflammatory response, macrophage (plasticity) polarization, fungal melanin, yeast mannoprotein

## Abstract

Pathogenic eukaryotes including fungi release extracellular vesicles (EVs) which are composed of a variety of bioactive components, including peptides, nucleic acids, polysaccharides, and membrane lipids. EVs contain virulence-associated molecules suggesting a crucial role of these structures in disease pathogenesis. EVs derived from the pathogenic yeast phase of *Talaromyces (Penicillium) marneffei*, a causative agent of systemic opportunistic mycoses “talaromycosis,” were studied for their immunogenic components and immunomodulatory properties. Some important virulence factors in EVs including fungal melanin and yeast phase specific mannoprotein were determined by immunoblotting. Furthermore, fluorescence microscopy revealed that *T. marneffei* EVs were internalized by THP-1 human macrophages. Co-incubation of *T. marneffei* EVs with THP-1 human macrophages resulted in increased levels of supernatant interleukin (IL)-1β, IL-6 and IL-10. The expression of THP-1 macrophage surface CD86 was significantly increased after exposed to *T. marneffei* EVs. These findings support the hypothesis that fungal EVs play an important role in macrophage “classical” M1 polarization. *T. marneffei* EVs preparations also increased phagocytosis, suggesting that EV components stimulate THP-1 macrophages to produce effective antimicrobial compounds. In addition, *T. marneffei* EVs stimulated THP-1 macrophages were more effective at killing *T. marneffei* conidia. These results indicate that *T. marneffei* EVs can potently modulate macrophage functions, resulting in the activation of these innate immune cells to enhance their antimicrobial activity.

## 1 Introduction

Talaromycosis (penicilliosis) is an invasive mycosis caused by the thermally dimorphic fungus *Talaromyces marneffei*, formerly known as *Penicillium marneffei*. This fungus is endemic throughout southeast Asia (e.g. in northern Thailand, Vietnam, and Cambodia), east Asia (e.g. in the south of mainland China, Hong Kong, and Taiwan), and northeastern India (e.g. in the seven sister states such as Manipur, Assam and Nagaland) (1–3). *T. marneffei* has long been recognized as one of the most common opportunistic diseases in individuals with HIV, frequently ranking third (after tuberculosis and cryptococcosis) in these endemic areas (4). Talaromycosis has recently been increasingly identified in non-HIV infected patients with impaired cellular mediated immunity, such as systemic lupus erythematosus (SLE), hematologic malignancy, lymphoma, solid organ transplanted patients receiving immunosuppressive drugs, adult-onset immunodeficiency syndromes (AOID), and immune reconstitution inflammatory syndrome (IRIS) (5, 6). Recently, The World Health Organization (WHO) has indeed prioritized the issue of invasive fungal infections by releasing the first Fungal Priority Pathogen List (WHO FPPL) in 2022. This list is aimed at guiding research and resource allocation towards the development of new and improved diagnostic tools, treatments, and preventive measures for the most significant fungal pathogens. The realizing of *T. marneffei* in WHO FPPL indicates a significant level of public health impact and that there is a need for further investigation in various aspects, including improving the sustainability of fundamental knowledge of *T. marneffei* (7).

*T. marneffei* is an opportunistic airborne fungal pathogen that affects the respiratory system of patients. Infection is presumed to occur via inhalation of conidia, which then transform to yeast and spread from the lungs to other organs via the reticuloendothelial system (1). *T. marneffei* is classified as a facultative intracellular pathogen, and the phase transition into yeast occurs within phagosomes of macrophages after the phagocytosis of conidia (8). In order to survive and proliferate within macrophages, *T. marneffei* yeasts have established a variety of adaptation or attack strategies to enhance their pathogenicity and virulence in the hostile environment of the host (9).

The complete pathogenic processes of talaromycosis remain unclear. However, a variety of potential factors including adhesion to host tissues (10), dimorphic switching (11, 12), melanin production (13), thermotolerance (14), cell wall mannoproteins Mp1p (15), intracellular proteinases (16), iron acquisition and gluconeogenesis control (17) and extracellular vesicles (EVs) (18) have been investigated in relation to the pathogenesis of *T. marneffei*.

EVs are nanoparticles composed of lipid bi-layered membrane structures that contain diverse proteins, lipids, polysaccharides, nucleic acids, and pigments. They are crucial in cell-to-cell communication, physiology, and immunopathogenesis of fungal infections (19–21). Several pathogenic fungi such as *Cryptococcus neoformans* (22), *Paracoccidioides brasiliensis* (23), *Histoplasma capsulatum* (24), *Sporothrix schenckii* (25), and *Candida albicans* (26) produce and release EVs, which have been hypothesized as essential regulators of physio-pathological pathways during fungal infections. *T. marneffei* EVs have recently been examined for their function in immunologically active components and virulence factors of talaromycosis (18). EVs produced by *T. marneffei* enhanced the production of reactive oxygen species (ROS), nitric oxide (NO), and certain inflammatory mediators such as IL-1β, IL-6, and TNF-α in RAW 264.7 murine macrophage cells. However, this proinflammatory effect of EVs was diminished after EV proteins were digested (18). Potential virulence factors such as heat shock protein, mannoprotein 1, and peroxidase were also found in the proteome of *T. marneffei*-derived EVs.

Since EVs have the potential to contribute to the pathogenesis of many fungal infections, we examined the immunological effects of *T. marneffei* EVs on phagocytes and their ability to induce fungal clearance. Moreover, EVs from *T. marneffei* induced M1 classical polarization of THP-1 human macrophages, resulting in increased fungicidal activity. We also confirmed that *T. marneffei* EVs carry important virulence factors such as melanin and yeast phase specific mannoproteins that are involved in anti-fungal immunity and host pathogenicity.

## 2 Materials and methods

### 2.1 *T. marneffei* and growth conditions

*T. marneffei* ATCC 200051 was routinely sub-cultured on Potato Dextrose Agar (PDA; Difco, Sparks, MD, USA) at 25°C for 5-7 days as described (27). The conidial suspensions were prepared by gentle scraping with a sterile cotton swab to sterile phosphate buffer saline (PBS, pH 7.4). The resulting suspension was filtered with sterile glass wool to selectively eradicate the short hyphae. The conidial suspensions were quantified by counting with cell counting chamber (Fuchs-Rosenthal chamber) and kept at 4°C in PBS pH 7.4 for subsequent experiments.

### 2.2 Isolation of *T. marneffei* yeast phase extracellular vesicles

The isolation of EVs was carried out with the modified protocol described by Rodrigues et al. (22) and performed on ice or 4°C for prevent vesicle rupture or fusion (23). For yeast phase culturing, *T. marneffei* conidia (5x10^6^ conidia/ml) were cultured in 150 ml of brain-heart infusion broth (BHI: Difco, Sparks, MD, USA) and incubated on a shaking incubator at 150 rpm at 37°C for 7 days. Thereafter, 2 step centrifugation of fungal cell free supernatant at 5,000 rpm (30 min, 4°C) and 10,000 rpm (60 min, 4°C) were followed to ensure that smaller debris were removed. The fungal cell free supernatants were concentrated using 100 kDa molecular weight cut-off Vivaspin 20 concentrators (GE Healthcare, Uppsala, Sweden). The concentrated supernatants were again centrifuged at 10,000 rpm (30 min, 4°C) to remove aggregate substances. The resulting supernatant was ultracentrifuged at 200,000 g for 90 min by Optima MAX-XP ultracentrifuge (Beckman Coulter, Brea, CA, USA) to precipitate the EVs. The precipitated EVs were washed and suspended with PBS pH 7.4. The amount of precipitated EVs was determined the total protein concentration by the Bicinchoninic acid assay with standard bovine serum albumin (Pierce BCA Protein Assay, Thermo Scientific, Rockford, IL, USA) (18).

### 2.3 Preparation of *T. marneffei* cytoplasmic yeast antigen

*T. marneffei* yeast cell pellets collected after cultivation in BHI as above were inhibited the growth by suspension in 0.02% (v/v) of thimerosal (Sigma-Aldrich, St. Louis, USA) at room temperature for 24 hours. The preparation of *T. marneffei* cytoplasmic yeast antigen (TM CYA) was carried out as described (28). The total protein concentration was quantified by the Bicinchoninic acid assay with standard bovine serum albumin (Pierce BCA Protein Assay, Thermo Scientific, Rockford, IL, USA).

To determine the amount of yeast phase secreted protein contained in culture supernatant, culture supernatants were obtained by growing *T. marneffei* in BHI broth at 37°C, 150 rpm for 7 days as described above. After harvest the yeast cell pellets, the culture supernatant was concentrated by using 10 kDa molecular weight cut-off Vivaspin 500 concentrator (GE Healthcare, Uppsala, Sweden). The remaining supernatant was collected, and protein concentrations were determined by Bicinchoninic acid assay and then analyzed by SDS-PAGE.

### 2.4 Monoclonal antibodies purification

The Mab 4D1 and Mab 8D6 hybridoma clones were cultured in 5% (v/v) FBS-RPMI 1640 (Gibco). The Mab 4D1 (murine IgG) binds a novel *T. marneffei* yeast phase-specific mannoprotein antigen and the Mab (29, 30) was purified by HiTrap column protein G affinity chromatography (GE Healthcare) as described (30). In contrast, the HiTrap IgM affinity column (2-mercaptopyridine coupled to cross linking epharose) was chosen to purify Mab 8D6 (murine IgM), which is a Mab specific to melanin (31). All experiments were carried out as per the manufacturer’s instructions.

### 2.5 SDS-PAGE and immunoblotting

Twenty micrograms/lane of TM CYA, concentrated culture supernatant, or *T. marneffei* EVs were mixed with Laemmli sample loading buffer containing β - mercaptoethanol and heated in boiling water for 10 min. The mixtures were subjected to SDS-PAGE separation on a reducing pre-cast Novex WedgeWell 4–20% Tris-glycine gel (Invitrogen, Carlsbad, CA, USA). Protein bands were stained with Commassie InstantBlue (Expedeon, Cambridge, UK). For immunoblotting, the polyacrylamide gel containing separated polypeptides were transferred electrophoretically to the 0.45 µm nitrocellulose membrane (Amersham, Uppsala, Sweden). Immunoblotting was performed using the *T. marneffei* yeast phase specific mannoprotein MAb 4D1 and melanin binding MAb 8D6 as described (30).

### 2.6 Visualization of EVs internalized by THP-1 macrophage

The collected Evs were labelled with fluorescein isothiocyanate (FITC, Sigma-Aldrich, St. Louis, USA) at 50 µg/ml in 0.1M carbonate buffer (pH 9.0) at 4°C overnight with shaking, and then quenched by 10% bovine serum albumin (Sigma, St Louis, MO, USA) and washed three time with PBS and kept at 4°CC with the dark condition. The human monocytic leukemia cell line THP-1 (ATCC TIB-202) was routinely sub-cultured in RPMI 1640 medium (Gibco, Life Technologies, NY, USA) containing 10% (v/v) heat-inactivated fetal bovine serum (FBS) (Gibco, Life Technologies, NY, USA). For the induction of cellular differentiation to adhering macrophages, THP-1 cells (1×10^6^/ml) were seeded onto 6-well culture plates (Costar, Corning, NY, USA) covered with sterile coverslip in 1 ml of 10% (v/v) FBS -RPMI 1640 medium and 50 ng/ml of phorbol myristate acetate (PMA; *Sigma*, St Louis, MO, USA) for 48 hours (30). Control differentiated THP-1 cells were pretreated with 2 µg/ml of cytochalasin B (BioChemica, Darmstadt, Germany) for 2 hours at 37°C in 5% CO_2_ (32). Subsequently, FITC labeled Evs were suspended to incomplete RPMI-1640 and incubated with THP-1 cells for 4 h. The culture medium was then discarded, and the cells were fixed using 4% formaldehyde for 10 min at room temperature. After washing, THP-1 nuclei were stained with 4′,6-diamidino-2-phenylindole (DAPI, 0.5 μg/ml; Sigma-Aldrich, St. Louis, USA) in PBS for 5 min before washing in PBS. Images were acquired using a Nikon DS Fi1 fluorescence microscope.

### 2.7 Detection of THP-1 surface costimulatory molecules

To determine the polarization of THP-1 cells during exposure to *T. marneffei* EVs, the macrophage costimulatory surface molecules CD86 (M1 maker) and CD206 (M2 maker) were stained by indirect immunofluorescence procedures and analyzed by flow cytometry. In brief, THP-1 cells were treated with *T. marneffei* EVs (20 µg/ml), and the treated THP-1 cells were harvested at 36 hours after EVs treatment. THP-1 Fc receptors (FcRs) were blocked by 1% (v/v) heat inactivated FBS in PBS on ice for 30 min. After washing with PBS, the THP-1 cells were stained with 10 µg/mL murine antibodies to human CD86 or CD206 (Invitrogen, Rockford, IL, USA) for 45 min. The purified MAb 18B7 (mouse IgG) was used as an IgG isotype control (33). The cells were washed once with PBS containing 1% (v/v) heat inactivated FBS. The cells were then incubated for 45 min in the dark on ice with 10 µg/mL Alexa Fluor 488-conjugated goat anti-mouse IgG (Invitrogen, Eugene, OR, USA). After washing, the cells were fixed with 2% paraformaldehyde in PBS for 15 min at room temperature. The cells were suspended in PBS containing 1% FBS and analyzed using a flow cytometer (Beckman Coulter, Suzhou, China) (27).

### 2.8 THP-1 cytokines measurements

Monolayers of differentiated THP-1 cells were washed three time in incomplete RPMI-1640, placed in medium supplemented with *T. marneffei* EVs (20 µg/ml) and incubated for 24 and 36 hours at 37°C with 5% CO_2_. As an infection control, THP-1 cells were infected with *T. marneffei* conidia at MOI 10 for 24 and 36 hours at 37°C (27). The culture supernatants were collected and kept at -20°C until the cytokine assays were performed. Culture supernatants were assayed for interleukin (IL)-10, IL-1β and IL-6 content by sandwich enzyme-linked immunosorbent assay (ELISA) according to the manufacturer’s protocol (BioLegend, San Diego, CA, USA). The experiments were performed in triplicates and then analyzed as described (30, 34).

### 2.9 Determination of phagocytosis by flow cytometry and fluorescence imaging

The internalization rates of *T. marneffei* conidia during interaction with THP-1 cells were determined according to the method of Oliveira et al. with minor modifications (35). Briefly, PMA treated THP-1 monolayers were stimulated for 48 hours with EVs at a final concentration 40 µg/ml. As a positive control, PMA treated THP-1 were activated with 100 ng*/*ml of lipopolysaccharides from *Escherichia coli* O111:B4 (Sigma, St Louis, MO, USA) and 20 ng*/*ml of recombinant human interferon*-*γ (ImmunoTools, Germany) (36). *T. marneffei* conidia were labelled with FITC (Sigma-Aldrich, St. Louis, USA) at 100 µg/ml in 0.1M carbonate buffer (pH 9.0) at 4°C for overnight with shaking (27, 30). After extensive washing with PBS containing 0.1% (v/v) Tween 20, labelled conidia were incubated with THP-1 macrophages for 4 hours and 6 hours at a 5:1 conidia/macrophage ratio, followed by extensive washing with PBS for removal of nonadherent conidia. Another round of washings, cells were labeled with 4′,6-diamidino-2-phenylindole (DAPI, 0.5 μg/ml; Sigma-Aldrich, St. Louis, USA) in PBS for 5 min before washing in PBS. The phagocytosed THP-1 cells were detached from culture plates using a cell scraper. The detached cells were fixed with 4% formaldehyde (Sigma-Aldrich, St. Louis, USA) and washed. Finally, the cells were suspended in chilled PBS containing 1% (v/v) FBS (Gibco, Life Technologies, NY, USA) and analyzed using a flow cytometer (Beckman Coulter, Suzhou, China)

To visualize the internalization rate of THP-1 cells corresponding with a flow cytometry, THP-1 monolayers were seeded at 1x10^6^ cells/well in 6 well plate containing sterile coverslips and were differentiated as described above. THP-1 cells internalized with FITC-labeled *T. marneffei* conidia were fixed with 4% formaldehyde and then the THP-1 nuclei were stained with DAPI. Images were acquired using a Nikon DS Fi1 fluorescence microscope.

### 2.10 Fungicidal (killing) activity of THP-1 stimulated with EVs

PMA treated THP-1 monolayers were stimulated for 48 hours with Evs at a final concentration 40 µg/ml. As a positive control, PMA treated THP-1 were activated with 100 ng*/*ml of lipopolysaccharides from *Escherichia coli* O111:B4 (Sigma, St Louis, MO, USA) and 20 ng*/*ml of recombinant human interferon*-*γ (ImmunoTools, Germany). Subsequently, the supernatants were aspirated, and the monolayers washed with incomplete RPMI 1640. Aliquots of *T. marneffei* conidia suspended in incomplete RPMI 1640 were added to the macrophages at MOI 5:1 conidia/macrophage ratio. After incubation for 2 hours at 37°C with 5% CO_2_, the samples were washed three times with incomplete RPMI 1640 to remove nonadherent conidia and fresh medium was added. The cells were incubated for 24 hoursunder the conditions described above. Next, cells were trypsinized, washed, and then lysed with 1% triton x-100 in PBS. THP-1 lysates containing fungal cells were immediately inoculated onto PDA at 25°C and CFUs were enumerated by 48 hours. As a control, unstimulated THP-1 (treated with PMA alone) were cultivated under the same conditions. The percentage of fungicidal activity (% killing) was calculated as described (34) using the formula:


$$\%\text{Fungicidal (killing) activity}=\frac{\left[1-\text{CFU Test}\right]}{\left[\text{CFU control }(0\text{ hour})\right]}$$

### 2.11 Transmission electron microscopy

Transmission electron microscopy (TEM) was performed to illustrate the intracellular structure of *T. marneffei* within THP-1 macrophages. Monolayers of differentiated THP-1 cells (1×10^6^ cells/well) in 6 well tissue culture plates were infected with *T. marneffei* conidia at MOI 10:1 conidia/macrophage ratio. After 36 hours of co-incubation at 37°C with 5% CO_2_, THP-1 infected with *T. marneffei* were removed by trypsinization, neutralized by completed RPMI-1640 and washed three times with PBS (27). Subsequently, intact *T. marneffei* infected THP-1 cells were fixed with 2.5% glutaraldehyde in 0.1M phosphate buffer and post fixed with 2% osmium tetroxide in PBS for 1 hour. Next, the samples were dehydrated with graded ethyl alcohol series, starting with 50, 70, 85, 95% and three exchanged with anhydrous absolute ethyl alcohol in the final step and then embedded in Epon-812 epoxy resin. The samples were then polymerized in a 60 °C in hot air oven for approximately 72 hours and ultrathin sections were cut at a thickness of 60-90 nm. The processed samples were mounted with uranyl acetate and viewed in a JEOL JEM- 2010 transmission electron microscope (JEOL, Tokyo, Japan), with an accelerating voltage 200 kV.

### 2.12 Statistical analysis

The data was subjected to statistical analysis using either an Ordinary one-way ANOVA and Turkey’s multiple comparison test, or a two-way ANOVA and Sidak’s multiple comparisons test, depending on the experiment. A significant value of *p*< 0.05 was used for all analyses. GraphPad Prism 7.0 software (version 7.0) and the QuickCalcs tool available on the GraphPad website (https://www.graphpad.com/quickcalcs/) were used for all statistical analyses.

## 3 Results

### 3.1 Identification of yeast phase specific mannoprotein and melanin in *T. marneffei* EVs

**Figure 1:**
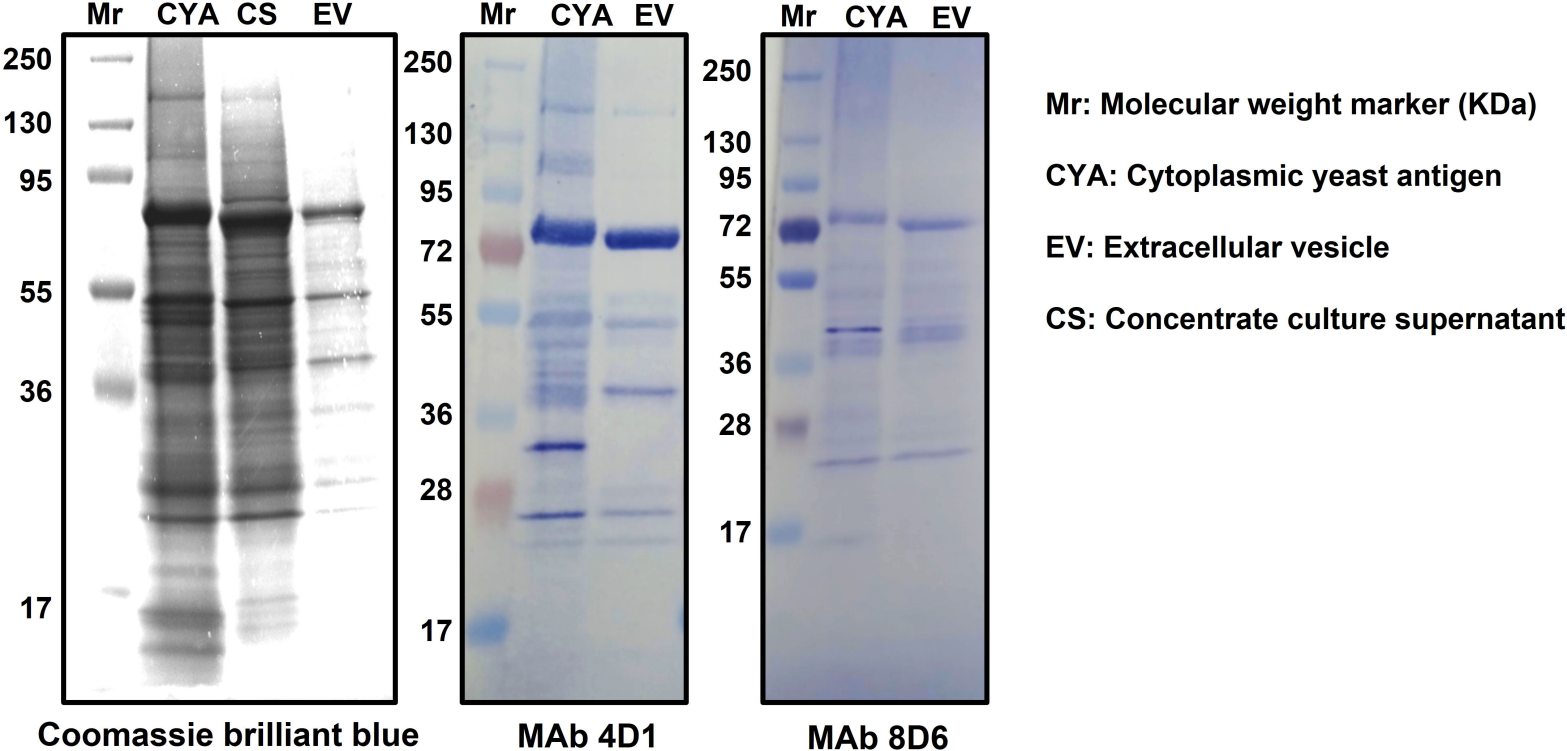
Specific recognition of immunogenic associated molecules within *T. marneffei* EVs by MAb4D1 and MAb8D6. The biochemical characterization of the composition of *T. marneffei* EVs using SDS-PAGE 4-20% gradient gel and immunoblotting showed that the profiles of the vesicles were similar to those of the cytoplasmic yeast antigen (CYA). The observation that both MAb 4D1 (anti-yeast phase specific mannoprotein) and MAb 8D6 (anti-melanin) recognized multiple undefined bands in the vesicles, with the greatest intensity observed against a peptide with a molecular weight approximately 72 kDa, suggests that these monoclonal antibodies are binding to specific epitopes present in the yeast phase of *T. marneffei* EVs.

The composition of *T. marneffei* cytoplasmic yeast antigen (TM CYA), culture supernatant and EVs were analyzed using SDS-PAGE gradient gels and immunoblotting using MAb 4D1 to *T. marneffei* yeast phase specific mannoprotein and melanin-binding MAb 8D6. The SDS-PAGE profiles of TM CYA, concentrate supernatant and EVs preparation were similar, with the visible components migrating within a molecular weight of 17 – 130 kDa. For immunoblotting, TM CYA from *T. marneffei* yeast was used to compare with the composition of EVs. We analyzed the ability of MAb 4D1 and MAb 8D6 to bind to TM CYA and EVs. Both antibodies recognized multiple undefined bands with molecular masses exceeding than 28 kDa. The greatest intensity was observed against a peptide with molecular weight of 72 kDa and a smear band between 55 and 36 kDa (**Figure 1**). This provides the first evidence that the epitopes recognized by MAb 4D1 and MAb 8D6 are present in *T. marneffei* EVs and, the immunoreactivity profiles of both antibodies against EVs are similar to the reactivity against TM CYA.

### 3.2 *T. marneffei* EVs were internalized by THP-1 macrophages

**Figure 2:**
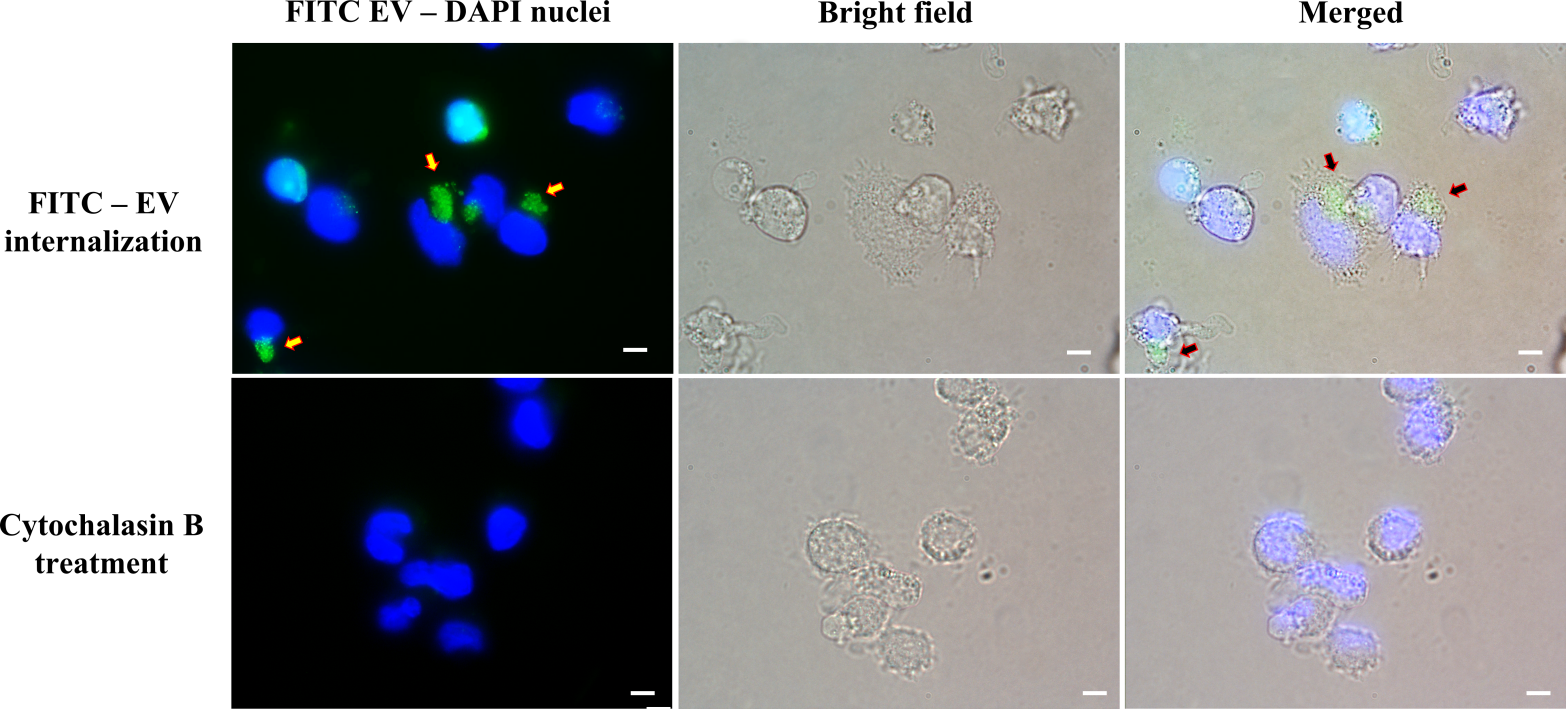
Fluorescence imaging revealed the activity of THP-1 macrophages internalizing *T. marneffei* EVs. THP-1 cells were co-incubated with FITC-labeled vesicles for 4 hours. The arrowheads indicate groups of FITC-labeled vesicles (Upper right panel). THP-1 cells were incubated with the actin polymerization inhibitor “cytochalasin B” before the process of FITC-labeled vesicles internalization. THP-1 nuclei were stained blue with DAPI. The photographs were taken between the fluorescence and bright field channels under 1,000x magnifications with a Nikon Eclipse 50i fluorescence microscope. The scale bars represent 5 µm.

The efficacy of THP-1 macrophages in internalizing *T. marneffei* EVs was examined by FITC labelled EVs fluorescence microscopy. *T. marneffei* EVs rapidly entered into THP-1 macrophages (**Figure 2**). The use of mycotoxin “cytochalasin B,” which disrupts the network formation by THP-1 F-actin filaments, decreased EVs endocytosis compared with the non-treatment group after 4 hours of incubation with the macrophages, indicating the important role for F-actin filaments in this process (37). These results suggest that the uptake of EVs by THP-1 macrophages is dependent on the presence of intact actin filaments and may involve both passive diffusion and active transport mechanisms (18).

### 3.3 *T. marneffei* EVs induced proinflammatory cytokine production

**Figure 3:**
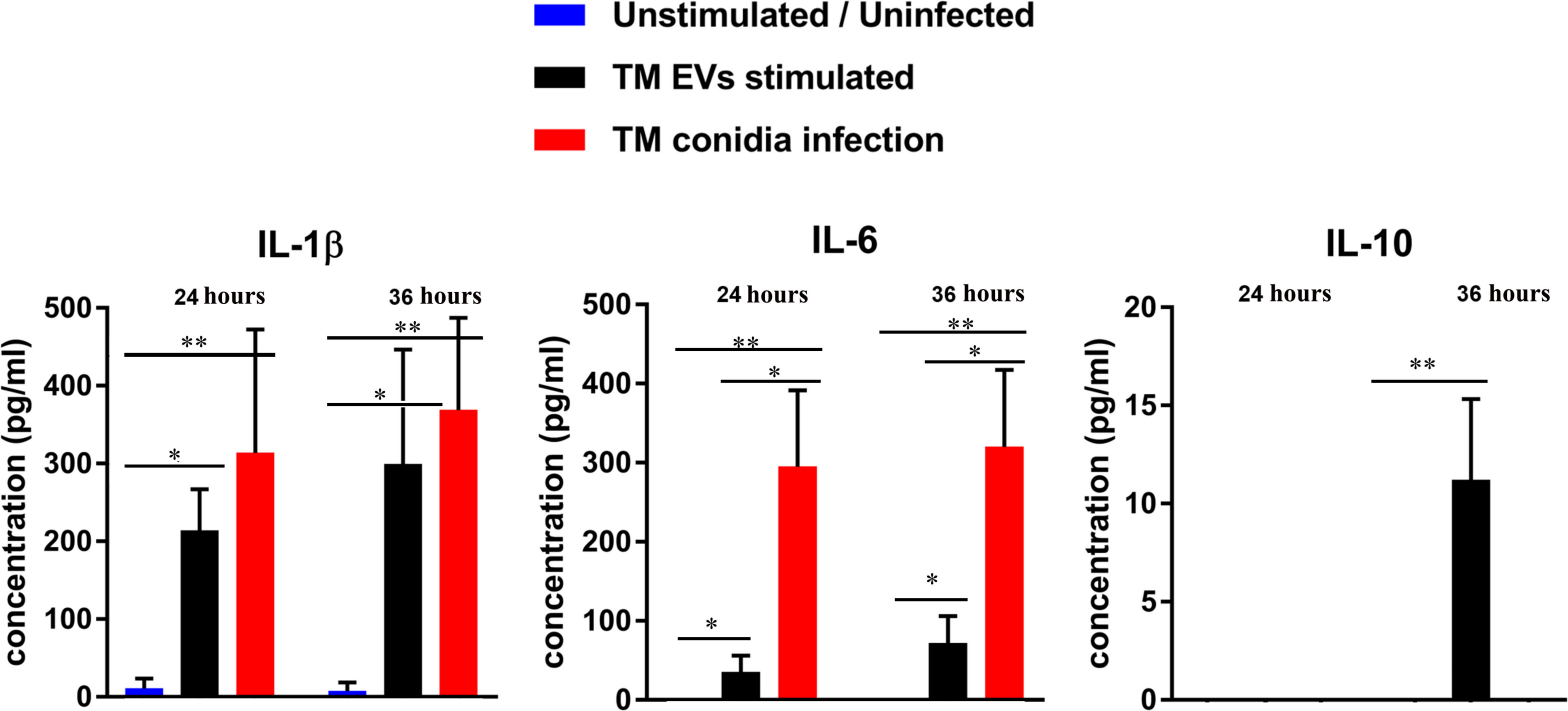
The cytokine profiles of THP-1 macrophages after co- incubation with *T. marneffei* EVs or *T. marneffei* conidia. IL-1β, IL-6 and IL-10 levels in the culture supernatants were determined by sandwich ELISA. THP-1 macrophages were stimulated with either *T. marneffei* EVs or *T. marneffei* conidia. The basal levels (PMA treated, but unstimulated or uninfected THP-1; blue bars), EVs stimulation (black bars) and *T. marneffei* conidia infection (red bars) of each cytokine are shown. The asterisks indicate statistical significance (*p< 0.05, **p< 0.01) based on unpaired Student’s t-test. The error bars denote standard deviations of three independent measurements. (TM EVs; *T. marneffei* extracellular vesicles).

The responses of THP-1 macrophage cells to stimulation with *T. marneffei* EVs were determined by evaluation of their ability to secrete cytokines into the supernatant. THP-1 cytokine responses were compared between macrophages exposed to *T. marneffei* EVs, co-cultured with *T. marneffei* conidia (MOI 10) and PMA treated (control cells) at 24 and 36 hours. Exposure to *T. marneffei* EVs or *T. marneffei* infection induced a significant increase in pro-inflammatory mediators (IL-1β and IL-6) compared to unstimulated cells in a time-dependent manner (**Figure 3**). In contrast, anti-inflammatory IL-10 was slightly increased at 36 hours after exposure to *T. marneffei* EVs whereas the IL-10 levels for *T. marneffei* conidial infection were undetectable in our system (**Figure 3**). These observations correlated with the capacity of other exosome or EVs from several fungal pathogens to promote a pro-inflammatory response in host macrophages (18, 38, 39).

### 3.4 *T. marneffei* EVs modulated THP-1 macrophages M1 (classical) polarization

**Figure 4:**
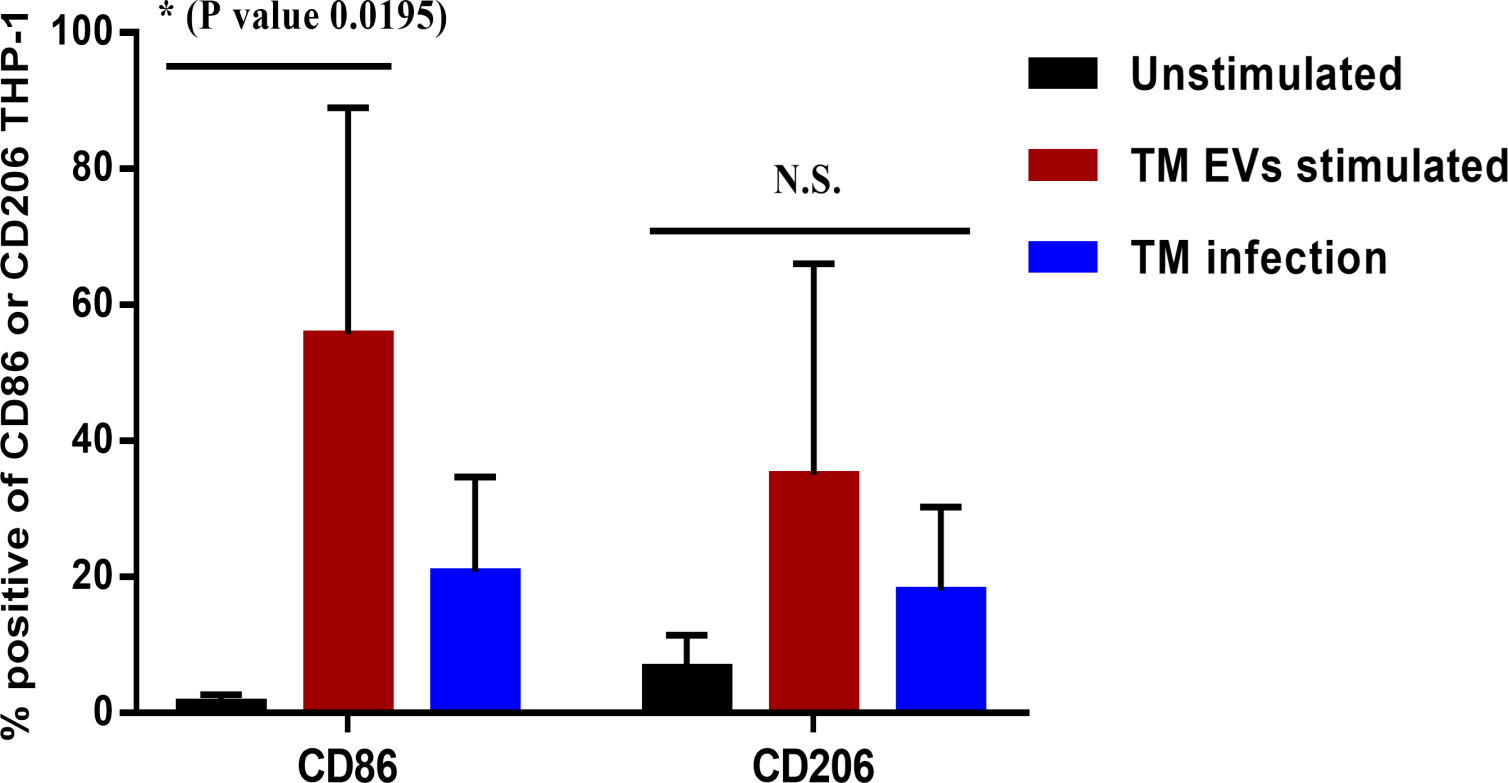
The percentages of THP-1 macrophage surface markers CD86 or CD206 after stimulation with *T. marneffei* Evs. After 36 hours of stimulation with *T. marneffei* Evs, THP-1 macrophages were stained with mouse anti-human CD86 or CD206 antibodies and a corresponding conjugate Alexa Fluor 488 labelled goat anti-mouse IgG antibody. The percentages of CD86^+^ or CD206^+^ macrophages were quantified by flow cytometer. The asterisks indicate statistical significance (*p< 0.05). The error bars denote standard deviations of three independent measurements. (N.S., Not statistically significant; TM Evs, *T. marneffei* extracellular vesicles).

The tendency to produce pro-inflammatory cytokines (IL-1β and IL-6) suggests that *T. marneffei* Evs favors the development of macrophages toward the “classical” M1 polarization (39, 40). To confirm the polarization of THP-1 macrophages during exposure to *T. marneffei* Evs, we measured the percentage of surface CD86 (M1 marker) and CD206 (M2 marker) expression by immunofluorescence staining and flow cytometry. When compared to unstimulated THP-1 (PMA treated), the expression of CD86 significantly increased. In contrast, the expression of CD206 also increased but this was not statistically significant. There was not statistically significance difference for the *T. marneffei* infection controls for both CD86 and CD206 staining. These findings support the hypothesis that exposure to *T. marneffei* Evs plays an important role in THP-1 macrophage “classical” M1 polarization (**Figure 4**).

### 3.5 *T. marneffei* EVs increase the phagocytic and fungicidal activity of THP-1 macrophages

**Figure 5:**
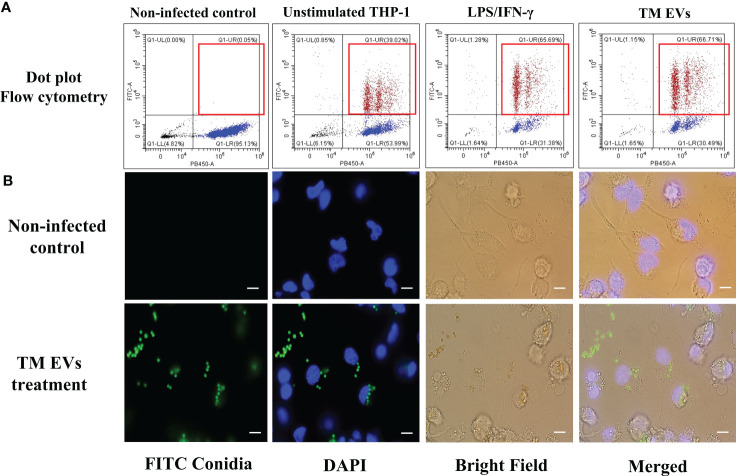
THP-1 macrophages stimulated with *T. marneffei* EVs exhibit enhanced phagocytic activity. Differentiated THP-1 macrophages, treated with *T. marneffei* EVs, LPS/IFN-γ, or control unstimulated THP-1 were infected with FITC-labelled *T. marneffei* conidia (green) and THP-1 nuclei were stained with DAPI (blue). The double positive signal in the dot plot (Q1-upper right, red squares) were quantified by flow cytometry **(A)**. THP-1 cells infected with FITC-labelled *T. marneffei* conidia, corresponding visualization by fluorescence microscopy of the material were prepared for analysis by flow cytometry. THP-1 nuclei were stained with DAPI. The photograph was taken between the fluorescence and bright field channels under 1,000x magnifications with a Nikon Eclipse 50i fluorescence microscope. The scale bars represent 5 µm **(B)**.

**Figure 6:**
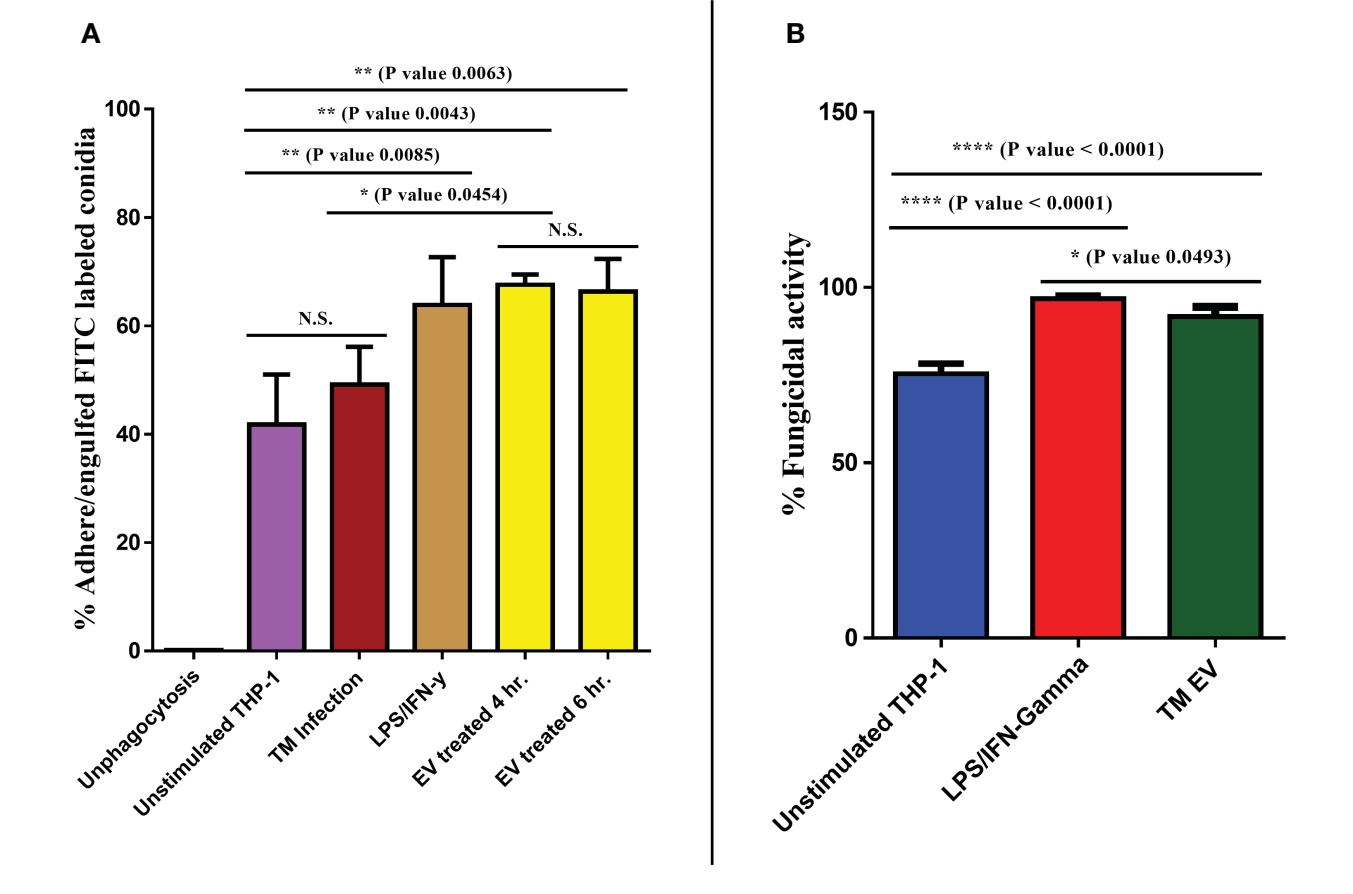
*T. marneffei* EVs promoted the phagocytic and fungicidal activities of THP-1 macrophages. To determine phagocytosis rates, the percentages of *T. marneffei* FITC-labelled conidia associated with macrophages were measured by flow cytometry **(A)**. To assess fungal killing, CFU determinations of THP-1 macrophages infected with *T. marneffei* conidia for 24 hours were performed. The normalized results (percentage of fungicidal activity) were compared to those obtained from unstimulated THP-1 macrophages (control) **(B)**. The asterisks denote statistical significance based on one way ANOVA. The error bars show standard deviations of three independent measurements. (N.S., Not statistically significant). (*p <0.05, **p <0.01, ****p <0.0001).

Since *T. marneffei* EVs stimulated THP-1 macrophages to produce increased amounts of pro-inflammatory cytokines and induced M1 polarization, we investigated whether EVs exposure could enhance THP-1 phagocytic and fungicidal activities to *T. marneffei* conidia. THP-1 macrophages previously treated with LPS/human recombinant IFN-γ, *T. marneffei* infection, *T. marneffei* EVs, or the unstimulated control (PMA treated) were co-cultured with FITC- labeled *T. marneffei* conidia for 4 or 6 hours. Flow cytometry of these cultures demonstrated the positive signals from macrophages containing FITC- labeled conidia and DAPI THP-1 nuclei staining. This double positive phenomenon indicated successful phagocytosis (indicated by the red square of each Q1-upper right quadrant). In contrast, a single positive signal from DAPI alone indicates macrophages without internalized conidia (Q1-lower right quadrant) (**Figure 5A**). From the quantification of results from three independent experiments, we observed that 67.51% and 66.33% of THP-1 macrophages activated with *T. marneffei* EVs displayed double positive signals at 4 h and 6 h of inoculation, respectively. Moreover, 63.87% of THP-1 macrophages were positive for FITC and DAPI staining after exposure to LPS/human recombinant IFN-γ and 49.14% of THP-1 macrophages were positive for *T. marneffei* infection control. In contrast, only 43.78% of unstimulated THP-1 cells (PMA treated) displayed double positive signals (**Figures 5A**, **6A**). The corresponding fluorescence imaging suggested that the FITC-labelled conidia were maximally internalized under the conditions used in our experiments. Confirming the results of flow cytometry, THP-1 macrophages stimulated with *T. marneffei* EVs had internalized FITC-labelled fungal conidia when compared to the uninoculated conditions (**Figure 5B**). *T. marneffei* infection control data was demonstrated in supplementary **Figure 1**.

Since phagocytosis and production of some antifungal phenotypes increased after exposure with *T. marneffei* Evs, we evaluated the fungicidal (killing) activity of THP-1 macrophages after exposure to Evs against *T. marneffei* conidia. Controls included unstimulated THP-1 (PMA treated) and activated THP-1 (treated with LPS/human recombinant IFN-γ). After 24 hours of inoculation of conidia with THP-1, subsequent CFU determinations demonstrated pretreatment with *T. marneffei* EVs killed 91.64% of conidia and stimulation with LPS/IFN-γ reduced conidia by 96.86% whereas unstimulated THP-1 were only able to kill 75.40% of the fungal cells (**Figure 6B**). Our findings indicate that *T. marneffei* EVs are potently effective at enhancing the fungicidal capacity of THP-1 macrophages, comparable to those stimulated by control LPS/IFN-γ (41).

### 3.6 EV-like structures were formed during *T. marneffei* replication inside THP-1 macrophages

**Figure 7:**
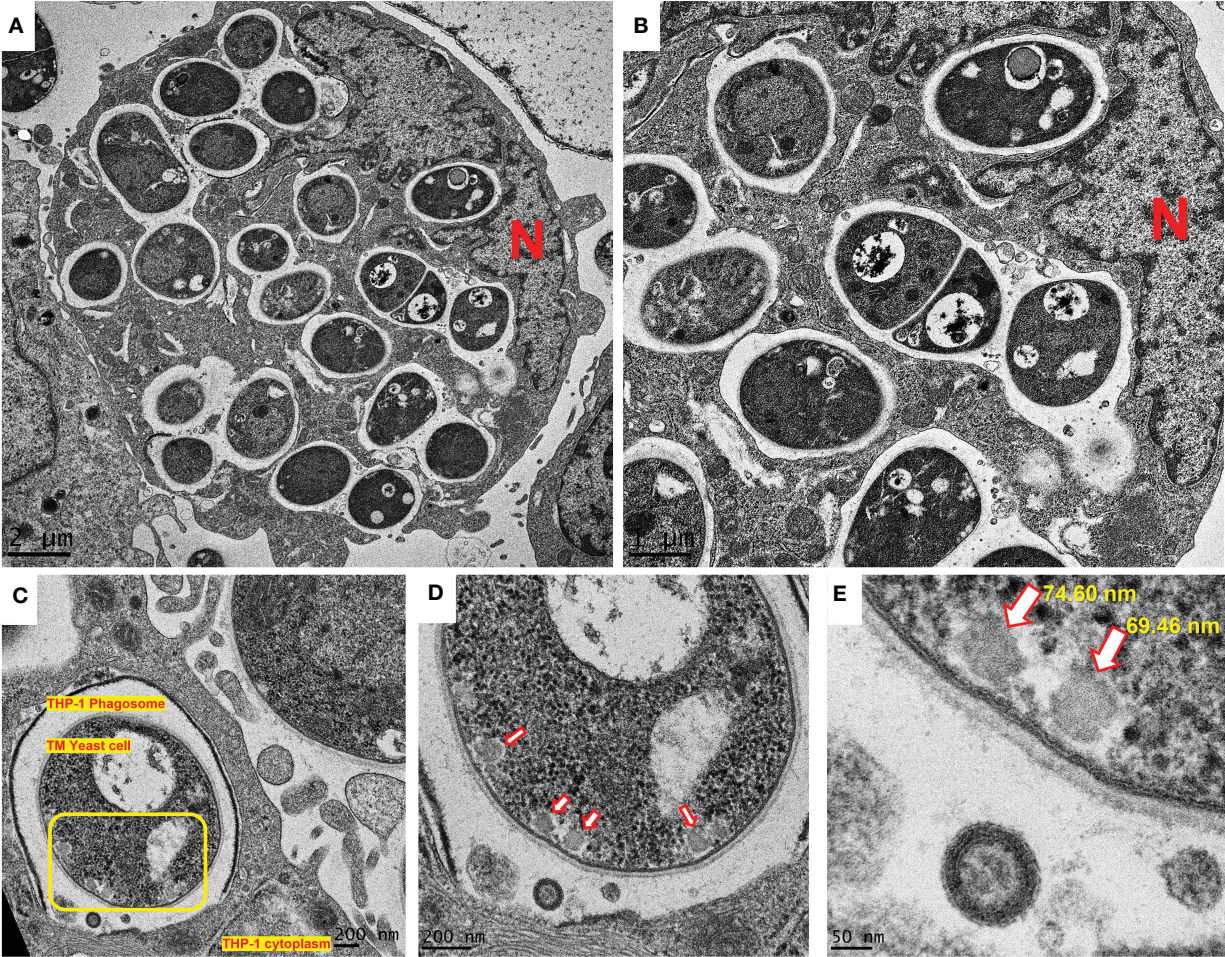
EV-like structures in T. marneffei yeast cells during morphogenesis and replication inside macrophages. The observation of EV-like structures in T. marneffei cells within the THP-1 human macrophages is a new discovery and suggests that this fungus utilizes EVs as a mechanism for modifying the host environment and promoting its survival. T. marneffei conidia undergo morphogenesis to fission yeast with a central transverse septum **(A, B)**. T. marneffei cells inside a macrophage phagosome are shown that reveal EV-like structures within the T. marneffei cytoplasm **(C)**. The presence of four EV-like structures were observed (indicated by red arrows, **(D)**. The measurement of the average size of these EV-like structures at 73 nm is consistent with a previous report (18) **(E)** and provides further evidence for the existence of these structures in T. marneffei yeast cell during replication inside macrophages. The photographs were taken with a JEM-2200FS transmission electron microscope. N represents the THP-1 nucleus. The scale bars (in µm or nm) are indicated on each figure. (TM, T. marneffei).

The ability to shift from conidia to yeast is a key aspect of *T. marneffei* pathogenicity, as it allows this organism to evade host defenses and establish infection (8). Conidia to yeast transition of *T. marneffei* inside THP-1 macrophage was demonstrated by transmission electron microscopy (TEM). TEM of a THP-1 human macrophage infected with *T. marneffei* demonstrates that *T. marneffei* conidia undergo morphogenesis to fission yeast with a central transverse septum (**Figures 7A, B**) within 36 hours of internalization. Representative *T. marneffei* cells inside a macrophage phagosome are shown that reveal EV-like structures in the *T. marneffei* cytoplasm, located near the surface of the cell wall (indicated by a yellow square, **Figure 7C**). When adjusting to a higher magnification, 4 additional EV-like structures are observed (indicated by 4 red arrows, **Figure 7D**). The dimensions of the EV-like structures were measured by software connected to the TEM (Gatan Digital Micrograph). The average size was quantified to be approximately 73 nm (**Figure 7E**), consistent with a previous report indicating the distributed size of *T. marneffei* EV as 30-300 nm (18). This is the first evidence to illustrate *T. marneffei* forming EV-like structure during infection within THP-1 human macrophages.

## 4 Discussion

EVs released by host cells infected by various microbes contain inflammatory mediators that can attract and activate host immune cells to promote or suppress the elimination of the invading pathogen (38, 41, 42). Fungi produce EVs as a strategy for transporting large molecules, including virulence factors, to the extracellular environment via the cell wall. Components of EVs derived from a variety of human pathogenic fungi can be detected by the host and its immune system, and EVs can alter and modulate immune responses (19). Thus, EVs released directly by the pathogens themselves also play an important role in modulating host innate immunity. These EVs contain bioactive components that can act as major drivers of the host immune response.

Recent research has demonstrated the presence of EVs in the yeast phase of *T. marneffei in vitro* (18). In our study, we report that EVs derived from *T. marneffei* have a role not only in modifying virulence strategies but also in modulating host immune responses. For virulent molecules, EVs can affect host anti-microbial activities by triggering the production of increased levels of cytokines, among other modifications (43). In the study by immunoblotting, *T. marneffei* EVs delivered fungal melanin and yeast phase specific mannoproteins, which were identified by anti-melanin MAb 8D6 and MAb 4D1, respectively. *T. marneffei* EVs contain immunogenic mannoproteins, which were recently described by proteomic analysis (18), and our result demonstates the presence of a phase specific mannoprotein (30). *C. neoformans* produces EV-associated DOPA melanin (44). Melanins play a role in the virulence of many pathogenic fungi, allowing them to thrive in harsh environments and enhance their resistance to host immune responses (45). Melanin serves as a powerful protector for *T. marneffei in vitro* by inhibiting phagocytosis and enhancing resistance to macrophage intracellular digestion (13). Furthermore, melanin-deficient mutants are more susceptible to antifungals, H_2_O_2_, and sodium dodecyl sulfate (SDS) when compared to the wild type (34). Woo et al. (2010) (46) have demonstrated that knocking down the melanin-biosynthesis gene cluster, *alb1*, in *T. marneffei* results in a reduction of virulence in mice when compared to the wild type. When exposed to hydrogen peroxide, the mutant conidia survival was 50% less than the wild type (46). Indeed, melanin has been termed “an antifungal resistance factor” due to its propensity to make melanized cells more resistant to antifungal agents (47) and this is supported by our prior study showing that melanin protects *T. marneffei* by making it more resistant to antifungal drugs (48). The presence of melanin in an immunoblotting by MAb 8D6 was interesting. It might be possible that melanin fragments were linked to proteins (melanoprotein), mannoproteins or melanosomes (49). In future studies, the large batch collection of *T. marneffei* EVs should be investigated with immunogold electron microscopy (IEM) techniques with the MAb 8D6. Altogether, our findings show that *T. marneffei* exports melanin and yeast phase specific mannoproteins as components of these “virulence factor delivery bags” that interact with host cells and modify innate immune responses. Additional important EV cargo molecules such as heat shock protein (HSP), catalase-peroxidase and another mannoprotein forms carried by *T. marneffei* EVs must be identified in future studies to clarify the potential functions of the EVs.

*T. marneffei* conidia first encounter the host innate immune system after inhalation of these fungal particles from the environment. The ability to shift from conidia to yeast is an important factor of *T. marneffei* pathogenicity, as it allows this organism to evade host defenses and establish infection (8). The host innate immune system plays a critical role in recognizing and eliminating fungal pathogens, and macrophages are one of the major cell types involved in this process. Macrophages are phagocytic cells that can engulf and destroy fungal cells through various mechanisms, including production of reactive oxygen species (ROS), lysosomal enzymes and the presentation of potent immunogenic peptide to specific helper T lymphocytes via the major histocompatibility complex (MHC) class II (50). The activation outcome of macrophages is polarized into 2 distinct phenotypes including M1 (protective) macrophages, which are associated with inflammation and microbe killing, characterized by the presence of pro inflammatory mediators. In contrast, M2 macrophages have non-protective immunoregulatory functions and are associated with the production of specific markers such as FIZZ-1, arginase-1, and chitinase-like YM1 (51). The switching between these phenotypes plays a critical role in the progression and control of fungal infection. In our study, *T. marneffei* EVs generated a significant level of pro-inflammatory cytokines (IL-1β and IL-6) and negligible amounts of IL-10 relative to unstimulated cells in a time-dependent manner. The plasticity of macrophage polarization during macrophage exposure to *T. marneffei* EVs has not been previously evaluated, and our data demonstrate the occurrence of THP-1 macrophage repolarization from M0 to classical M1 in response to *T. marneffei* EVs. Not only *T*. *marneffei* EVs but also other pathogenic fungal EVs, such as EVs from *P. brasiliensis*, *S. brasiliensis, Aspergillus flavus* and *T. interdigitale* exhibit a significant impact on classical M1 macrophage response (39, 40, 52, 53). Our study is consistent with previous descriptions by Yang et al. (18). The studies demonstrated that *T. marneffei* EVs induce the production of various cytokines, including IL-1β, IL-6, IL-10, and TNF-α by RAW264.7 murine macrophages. Moreover, there was upregulated the expression of CD80, CD86 and MHC class II in these macrophages. This evidence confirmed that *T*. *marneffei* EVs can modulate the host immune response and promote the production of pro-inflammatory cytokines and other inflammatory mediators such as reactive oxygen species (ROS) and nitric oxide (NO) (18). There are several reasons for which *T. marneffei* EVs might activate THP-1 macrophages extrapolated on LC–MS/MS by Yang et al. (18) First, activation of THP-1 pattern recognition receptors (PRRs); *T. marneffei* EVs contain pathogen-associated molecular patterns (PAMPs) e.g. β-glucans, mannans, and chitin (B6QEF2_TALMQ) or cell wall mannoprotein 1 (A0A455R7H9_TALMA) which recognize and activate PRRs on THP-1 cells. For example, β-glucans present in the fungal cell wall can activate the dectin-1 receptor on THP-1 cells, leading to the production of pro-inflammatory cytokines and pro-inflammatory mediators (54). Moreover, it may be possible that the melanin or melanoprotein inside the EVs can activate THP-1 macrophages by modulating toll-like receptor (TLR) 2 or TLR 4 on the THP-1 surfaces (55, 56). Second, delivery of bioactive compounds; *T. marneffei* EVs contain various bioactive compounds such as alkaline serine protease (B8M8A7_TALSN) or extracellular metalloprotease (A0A093UPI6_TALMA), which can directly damage host cells or modulate host immune responses. These effector molecules can activate THP-1 cells by inducing cytokine production or programed cell death (57). Overall, the mechanisms by which *T. marneffei* EVs activate THP-1 cells are complex and likely involve multiple pathways. The understanding of cellular and molecular level interactions between *T. marneffei* EVs and THP-1 cells will be investigated in future studies.

For phagocytosis and fungicidal assays, we used FITC-labelled *T*. *marneffei* conidia and flow cytometry to quantify the interaction of the *T*. *marneffei* conidia with THP-1 macrophages. The proportion of macrophages that adhered or ingested FITC-labelled conidia was equal to THP- macrophages stimulated with the positive control LPS/IFN-γ. These results exhibited that the M1 polarization of macrophages was induced by *T*. *marneffei* EVs and influenced the internalizing of the conidia. Remarkably, THP- 1 stimulated with EVs showed significantly enhanced intracellular killing of *T*. *marneffei*, as demonstrated by CFU determinations. This finding is consistent with the fact that RAW264.7 macrophage stimulated with *T*. *marneffei* EVs induced the generation of NO, IFN-γ and other phagocyte-derived anti-microbial compounds (18, 58). All together, these results show that *T*. *marneffei* EVs not only promote the internalization of *T*. *marneffei* conidia by THP-1 macrophages, but also enhance the fungicidal activity of such macrophages. This is consistent with reports for EVs from *C. neoformans*, *C. albicans*, *T. interdigitale* and *P. brasiliensis* that also promote the fungicidal activity of various types of macrophages (26, 35, 40, 52). It is worth noting that the study of *T. marneffei* EVs is still limited, and we cannot identify the definitive marker molecule that is specific for each type of EVs. Although we achieved to detected melanin (recognized by MAb 8D6) and yeast phase-specific mannoprotein (recognized by MAb 4D1) by using immunoblotting, but these proteins are some parts of the EV groups that are typically secreted from the fungal cell to the environmental outside (secreted form EVs). Moreover, the properties of proteins and biomolecules that compose or insert within EVs released from cells (secreted form EVs) and those inside cells (cytosolic form EVs) might differ. Previous studies reported that the composition of EVs released from *C. albicans*, *P. brasiliensis*, *H. capsulatum*, *Saccharomyces cerevisiae*, and *C. neoformans* contains glucan and/or chitin, which are important components found on the fungal cell wall. In contrast, EVs inside the cytoplasm have components derived from the plasma membrane (59).

**Figure 8:**
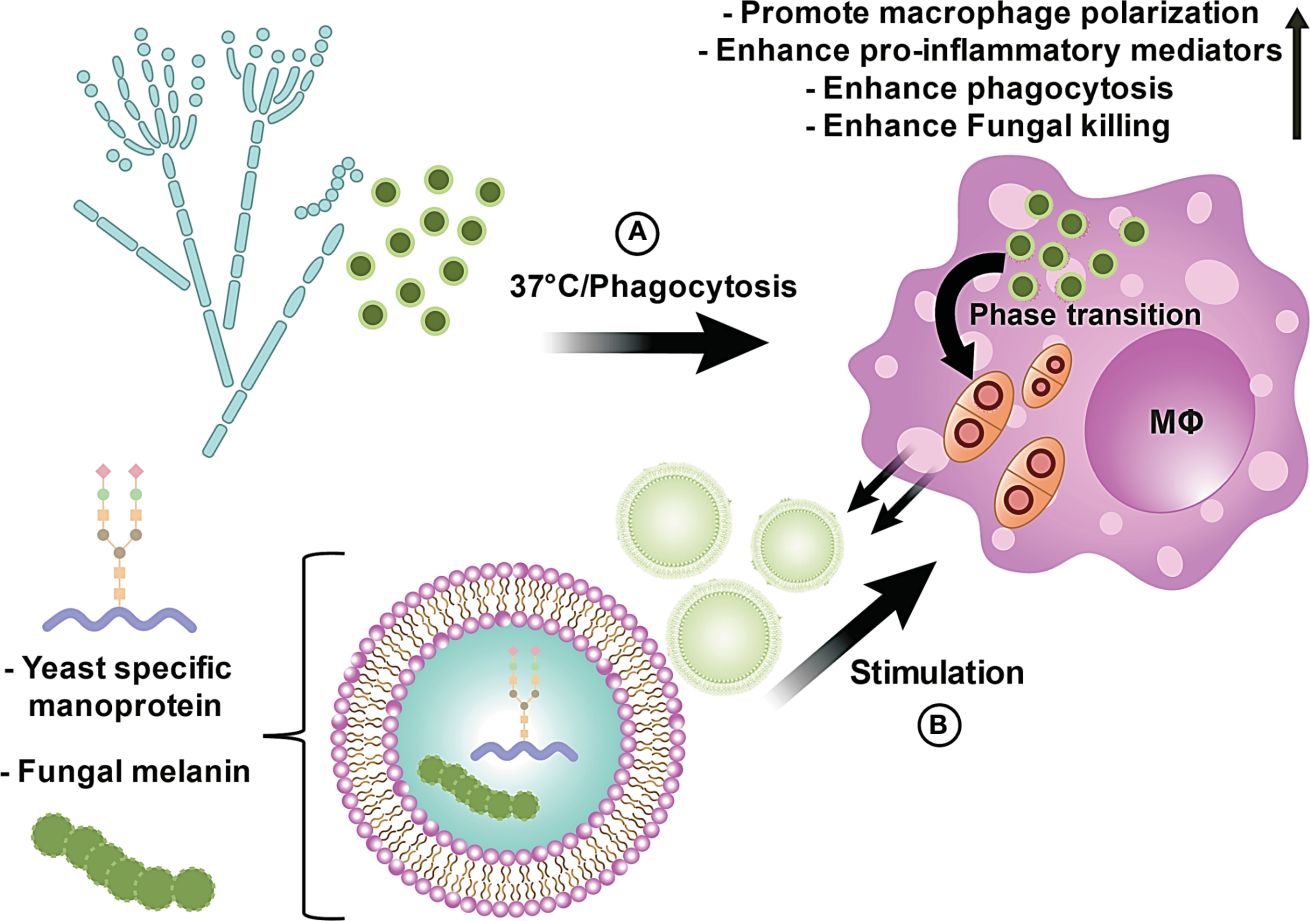
The proposed model illustrates the innate immunological effects of EVs from *T. marneffei* on THP-1 macrophages. **(A)** *T. marneffei* conidia are phagocytized by host macrophages and rapidly undergo morphogenic transition to the pathogenic yeast cells and multiply intracellularly inside host macrophages. **(B)** Co-incubation of THP-1 human macrophage-like cells with *T. marneffei* EVs loaded with important virulence factors, including melanin and yeast phase specific mannoprotein, induces THP-1 cell M1 polarization and increased release of pro-inflammatory cytokines. EV-treated M1 polarized THP-1 cells display increased phagocytic and fungicidal activities. Hence, EVs produced by *T. marneffei* yeast cells modify the host innate immune response.

In conclusion, we investigated the capacity of EVs derived from *T*. *marneffei* and found that these EVs deliver immunologically active compounds, including a yeast phase specific mannoprotein and melanin, to THP-1 macrophages in a process that modulates THP-1 macrophage polarization and enhances the phagocytosis and killing of *T. marneffei* conidia. We also demonstrated the generation of *T. marneffei* EVs during their intracellular phase transformation within THP-1 phagosomes (**Figure 7**). An illustration of these biological processes initiated by *T*. *marneffei* EVs on THP-1 macrophages is summarized in the proposed model depicted in **Figure 8**. Our findings provide new insights for further investigations into talaromycosis and introduce a novel target for the design of antifungal agents to combat these opportunistic mycoses.

## Data availability statement

The original contributions presented in the study are included in the article/**Supplementary Material**. Further inquiries can be directed to the corresponding author.

## Author contributions

KP, JN and SY conceived this project and supervised the experiments. KP, PT and SY designed research. KP, AA, and PT performed experiments. KP, AA, JN and SY analyzed experimental results. KP, AA, PT, JN and SY prepared the manuscript. All authors contributed to the article and approved the submitted version.
