# Supplementary material for: Extracellular vesicles derived from Talaromyces marneffei contain immunogenic compounds and modulate THP-1 macrophage responses
Source: Front Immunol. 2023 Jun 29;14:1192326. doi: 10.3389/fimmu.2023.1192326 (PMC10339390; doi:10.3389/fimmu.2023.1192326)
Supplement: Supplementary file 1 [file DataSheet_1.docx]

**Supplementary Figure 1**


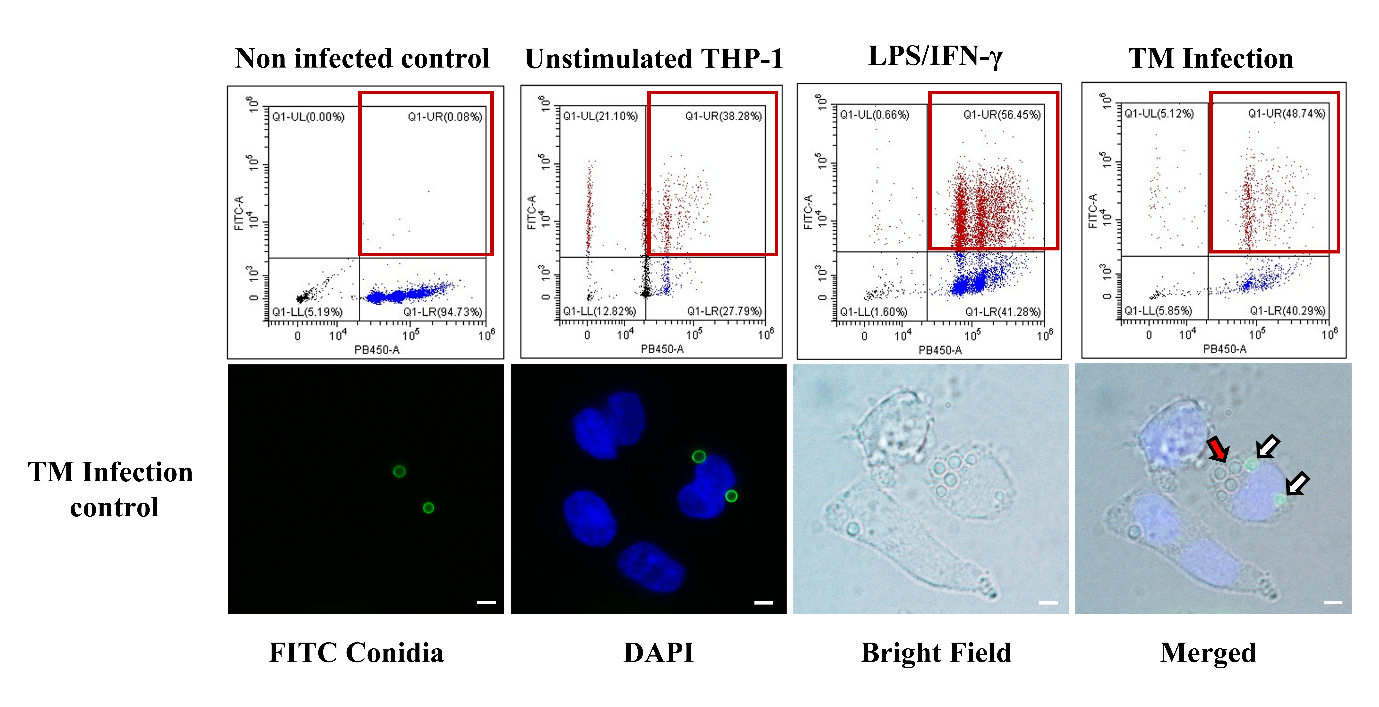


Differentiated THP-1 macrophages, infected with *T. marneffei* conidia (TM infection; Red arrow), LPS/IFN-γ, or control unstimulated THP-1 were phagocytosed with FITC-labelled *T. marneffei* conidia (green conidia; white arrow) and THP-1 nuclei were stained with DAPI (blue). The double positive signal in the dot plot (Q1-upper right, red squares) were quantified by flow cytometry.

THP-1 cells phagocytosed with FITC-labelled *T. marneffei* conidia, corresponding visualization by fluorescence microscopy of the material were prepared for analysis by flow cytometry. THP-1 nuclei were stained with DAPI. The photograph was taken between the fluorescence and bright field channels under 1,000x magnifications with a Nikon Eclipse 50i fluorescence microscope. The scale bars represent 5 µm.
